# Substituent Effects on the Photodeprotection Reactions of Selected Ketoprofen Derivatives in Phosphate Buffered Aqueous Solutions

**DOI:** 10.1038/srep21606

**Published:** 2016-02-22

**Authors:** Mingyue Liu, Ming-De Li, Jinqing Huang, Tianlu Li, Han Liu, Xuechen Li, David Lee Phillips

**Affiliations:** 1 Department of Chemistry, the University of Hong Kong, Pokfulam Road, Hong Kong

## Abstract

Photodeprotection is an important reaction that has been attracting broad interest for use in a variety of applications. Recent advances in ultrafast and vibrational time-resolved spectroscopies can facilitate obtaining data to help unravel the reaction mechanisms involving in the photochemical reactions of interest. The kinetics and reaction mechanisms for the photodeprotection reactions of ketoprofen derivatives containing three different substituents (ibuprofen, Br and I) were investigated by femtosecond transient absorption (fs-TA) and nanosecond time-resolved resonance Raman (ns-TR^3^) spectroscopy methods in phosphate buffered solutions (PBS). Fs-TA allows us to detect the decay kinetics of the triplet species as the key precursor for formation of a carbanion species for three different substituents attached to ketoprofen. To characterize the structural and electronic properties of the corresponding carbanion and triplet intermediates, TR^3^ spectroscopic experiments were conducted. The transient spectroscopy work reveals that the different substituents affect the photodecarboxylation reaction to produce carbon dioxide which in turn influences the generation of the carbanion species which determines the rate of the photorelease of the functional groups attached on the ketoprofen parent molecule. The fingerprint TR^3^ spectroscopy results suggest that ketoprofen derivatives may be deactivated to produce a triplet carbanion when increasing the atom mass of the halogen atoms.

Photoremovable protecting groups have received a great deal of attention^1^,^2^,^3^,^4^,^5^,^6^,^7^,^8^,^9^,^10^,^11^,^12^,^13^,^14^,^15^ due to their applications as powerful tools for the rapid introduction of different kinds of biological effectors into biological systems^1^,^6^,^7^,^10^ with spatial and temporal control which allows researchers to monitor the intermediate species by time-resolved methods^16^,^17^. Studies by Scaiano and co-workers have demonstrated that ketoprofen (KP), a non-sterioidal anti-inflammatory drug (NSAID) can be developed as a photodeprotection platform that can perform fast and efficient photorelease of functional groups after ultraviolet light excitation^18^,^19^. Much effort has been devoted to this KP based photodeprotection platform and it has been reported to be superior to some other photodeprotection compounds (o-nitrobenzyl group)^20^. For example, this photodeprotection platform denoted as “ketoprofenate derivatives” (KP-X) have many advantages such as cleanness and efficiency for release of the leaving group, good aqueous solubility and inert and non-absorbing photoproducts. Recently, KP-OAc, one of this new class of KP-X compounds has been used as a model to investigate the highly efficient phototrigger reaction in phosphate buffered aqueous solutions, (PBS) mediated by a carbanion intermediate but not taking place in an acid-assisted decarboxylation reaction^21^,^22^. However, the mechanism of ketoprofenate release for different substituent groups has not been so well reported and much work is still left to be done to explore how the nature of the leaving groups can influence the photodeprotection reactions of ketoprofenate derivatives.

In this work, we investigated the mechanisms of selected ketoprofenate phototrigger systems to release species like ibuprofen, bromide and Iodine (see compound structures in Fig. 1) by time-resolved spectroscopy in PBS with a pH value of 7.4. Femtosecond transient absorption spectroscopy (fs-TA) was employed to monitor the very early photophysical and photochemical processes after excitation of the samples by a 267 nm pump laser pulse. Nanosecond time-resolved resonance Raman (ns-TR^3^) spectroscopy experiments were carried out to detect the intermediates generated in the nanosecond to microsecond time range. It should be noted that ns-TR^3^ is a powerful technique that is able to obtain vibrational spectroscopic information that can help gain insight into the structure and properties of the intermediates observed. The rate constants and reaction mechanisms of these ketoprofenate phototrigger compounds were found to be greatly dependent on the different substituent in the PBS. Under ultraviolet laser irradiation, the KP-Br, I and ibuprofen photocages underwent intersystem crossing (ISC) with different time constants. Results of the fs-TA and ns-TR^3^ experiments indicated that the dynamics of each species can be well fitted and the critical intermediates (e.g. the carbanion and ketyl radical) can be reasonably assigned so as to develop a scheme for the photophysical and photochemical process involved in the photochemistry of interest (see mechanism proposed in Fig. 2). Finally, a clear and convincing mechanism can be concluded which is helpful for a better understanding of how these ketoprofenate phototrigger compounds work and may be useful in the design of new phototrigger compounds of NSAID in the future but also for related functional group delivery.

## Results

**Figure 3:**
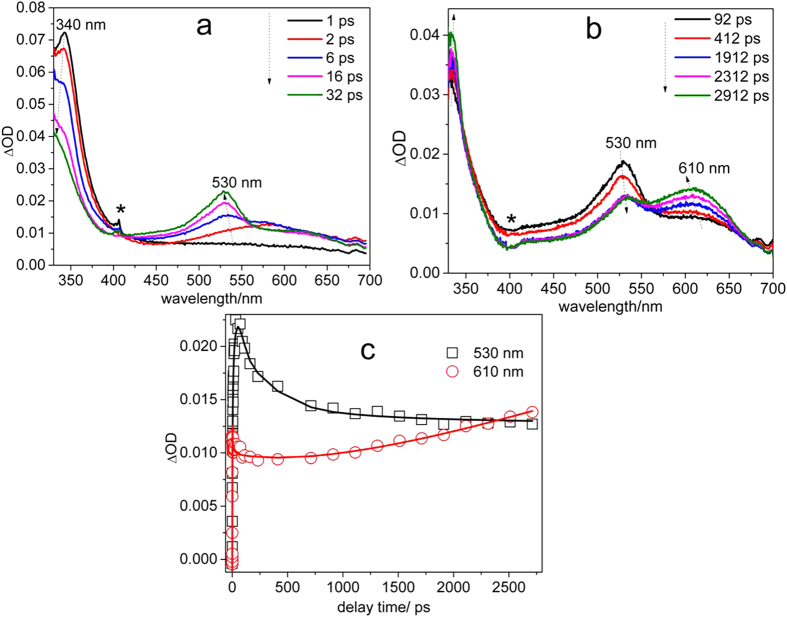
(**a**)Shown are fs-TA spectra of KP-ibuprofen in a pH = 7.4 PBS/MeCN = 1:1 by volume aqueous solution at early delay time and **(b)** late delay times excited by 267 nm and probed by a white light continuum. The asterisks (*) marks subtraction artifacts. **(c)** Shown are the temporal dependences of the transient absorption spectra at 530 nm and 610 nm for KP-iburprofen.

Fs-TA spectroscopy experiments were conducted to study the KP-ibuprofen, KP-Br and KP-I in phosphate buffered solutions (PBS): MeCN = 1:1 by volume mixed solution with a pH value of 7.4. Figure 3(a) displays the early delay time evolution of KP-ibuprofen in the PBS:MeCN = 1:1 by volume mixed solution from 1 ps to 32 ps. An absorption band at 340 nm gradually decays to 328 nm while another absorption band at 575 nm grows in and shifts to 530 nm. This process is associated with intersystem crossing (ISC) from S_1_ to T_1_ according to its similarity to results from previous fs-TA studies of ketoprofen derivatives^21^. Then the decarboxylation reaction takes place in the triplet state as is shown in Fig. 3(b) which demonstrates that the triplet with its maximum absorption bands at 530 nm and 328 nm decay while a longer wavelength broad absorption band at 610 nm grows in at the cost of triplet state from 92 ps to more than 2500 ps. The isosbestic point for this reaction clearly indicates that the KP-ibuprofen triplet anion is the precursor of the 610 nm species which is readily assigned to a KP carbanion species^21^. The corresponding kinetics of the transient absorption at 530 nm and 610 nm are shown in Fig. 3(c). The kinetics at 530 nm was fitted by a bi-exponential function with time constants of t_1_ = 9 ps and t_2_ = 555 ps. The t_1_ is associated with the ISC process and t_2_ is assigned to the time constant of the photo-decarboxylation process that converts the triplet into the KP carbanion species^21^,^23^. The kinetics of the growth at 610 nm was able to be fit by a time constant of 649 ps which is a reasonably consistent value when compared with that of the decay time of t_2_ 555 ps at 530 nm. This suggests that the KP carbanion species is formed from the triplet species. The deviation of the two time constants which differ by ~100 ps may be explained by the mutual uncertainty of the fits of the experimental data. Therefore, the KP-ibuprofen photocage behaves in a similar manner as that of KP in a PBS. Namely, it undergoes a photodecarboxylation reaction efficiently after photoexcitation.

**Figure 4:**
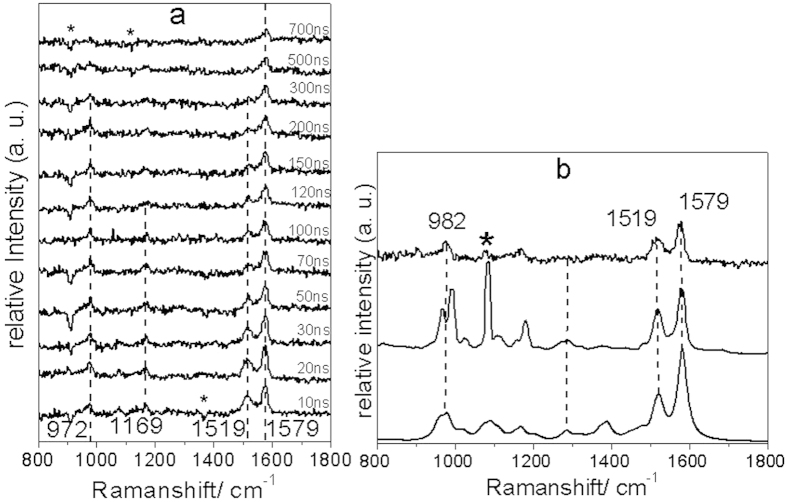
(**a**)Shown are the ns-TR^3^ spectra of KP-ibuprofen in a PBS/MeCN = 1:1 by volume solution pH = 7.4 obtained with a 266 nm pump excitation wavelength and a 319.9 nm probe wavelength at various delay times that are inserted next to the spectra. **(b)** Shown is a comparison of the experimental resonance Raman spectrum of KP-ibuprofen obtained in a MeCN/PBS = 1:1 (volume ration) solution at 20 ns delay time (top), the experimental resonance Raman spectrum of the ketoprofen biradical species at a delay time of 80 ns from a previous study (middle); and the DFT calculated normal Raman spectrum for the benzylic biradical (bottom). Dotted lines display the correlation between the experimental and calculated Raman bands. The asterisk (*) marks subtraction artifacts.

Figure 4(a) displays the ns-TR^3^ spectra obtained for KP-ibuprofen in a PBS: MeCN = 1:1 by volume mixed solution with pH = 7.4. Only one species was observed in the spectra throughout the delay times examined in the figure. This species which features intense Raman bands at 972, 1519 and 1579 cm^−1^ was assigned to a KP-ibuprofen biradical species which is a resonant structure of the triplet state KP-ibuprofen carbanion by protonation and deprotonation^23^. The carbanion species evolves at the very beginning of the delay time and decays within ~300 ns. The decay of the carbanion species indicates that KP-ibuprofen releases ibuprofen at the cost of the carbanion species similar to the way that KP-OAc releases OAc as reported in a previous study^21^. Figure 4(b) shows a comparison of a selected experimental resonance Raman spectrum at a delay time at 20 ns of KP-ibuprofen (top) from Fig. 4 (a) with the spectrum of the KP biradical species resonance Raman spectrum obtained from a previous study (middle) and to a Density Functional Theory (DFT) calculated normal Raman spectrum for the biraidcal structure intermediate (bottom). The good agreement of this comparison confirms that the second species is the biradical species from KP-ibuprofen. These results indicate that KP-ibuprofen underwent a decarboxylation reaction from its triplet state. The intensity of the Raman band at 1519 cm^−1^ decays quickly from 20 ns to 300 ns. This decay indicates that the carbanion drives the ibuprofen-anion off from its ketoprofen parent structure at the cost of the carbanion species.

**Figure 5:**
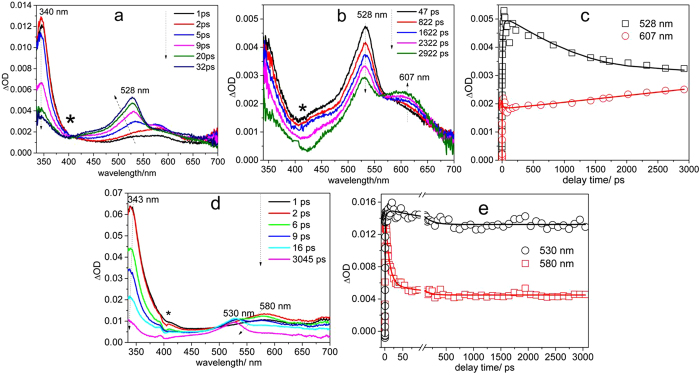
**(a)**Shown are the fs-TA spectra of KP-Br in a pH = 7.4 PBS/MeCN = 1:1 by volume aqueous solution at early delay times and **(b)** later delay times excited by 267 nm light and interrogated by a white light continuum probe pulse and **(c)** its corresponding temporal dependence of the transient absorption spectrum at 528 nm and 607 nm. **(d)** Shown are the fs-TA spectra of KP-I in a pH = 7.4 PBS/MeCN = 1:1 by volume aqueous solution at late delay times excited by 267 nm light and interrogated by a white light continuum probe pulse and **(e)** its corresponding temporal dependence of the transient absorption spectrum at 530 nm and 580 nm. The asterisk (*) marks subtraction artifacts.

To study substituent effects, halogen derivatives of KP were also investigated using the same methodology as the Ibuprofen substituent of KP using fs-TA spectroscopy and ns-TR^3^. KP-Br was observed to undergo a similar photodecarboxylation reaction with a lower quantum yield compared with the analogous photodecarboxylation reactions of KP-ibuprofen and KP-OAc as deduced from the relative low intensity of the absorption region indicated by the carbanion species intensity at 607 nm. Figure 5(a) presents the early delay time spectra of KP-Br from 1 ps to 32 ps. Like KP-Ibuprofen’s behavior, at early delay time, it partially underwent an intersystem crossing from the singlet to its triplet. Figure 5(b) displays the later time scale spectra for fs-TA from 47 ps to 2922 ps in a PBS:MeCN = 1:1 by volume mixed solution for KP-Br. The spectra show a similar behavior as that of KP-Ibuprofen. The absorption band at 528 nm is associated with triplet species which starts to decay and converts to a new species evolving at 607 nm which can be assigned as the KP carbanion species similar to that as KP-ibuprofen’s. The isosbestic point at 555 nm clearly demonstrates that the triplet is the precursor of the carbanion species whose absorption grows in at 607 nm. The transient absorption kinetics at 530 nm and 607 nm are given in Fig. 5(c) which found that the triplet decay time constant is 1094 ps while the growth time constant from the triplet KP-Br to its carbanion is up to several nanosecond time scale which may imply that the Br substituent affects the reactivity of the BP parent structure more than that of KP-ibuprofen’s case. The longer conversion time constant and the difference between the decay of the triplet and the growth of the carbanion intermediate may imply that the attached Br substituent affects the reactivity of the KP triplet state which may induce other simultaneous competing reactions such as a hydrogen abstraction reaction or others to take place. In addition, the intensity at 607 nm is lower than that for the corresponding absorption for the KP-ibuprofen molecule.

On the other hand, KP-I underwent predominantly an intersystem crossing (ISC) to its triplet state only and then slowly decayed in intensity but without any photodecarboxylation reaction taking place in a PBS. To demonstrate the preceding trends clearly, two separated groups of spectra for early and later delay times are shown for the KP-ibuprofen spectra examined. Figure 5(d) shows the fs-TA spectra evolution of KP-I in a PBS solution for delay time from 1 ps to 3045 ps. The KP-I behaves like KP from its partial excited singlet state and undergoes a conversion to the triplet state through ISC. In Fig. 5(d) the 343 nm and 580 nm absorption bands are associated with the singlet state. At the cost of the singlet absorption bands intensity the triplet species forms at 530 nm. The corresponding kinetics at 530 nm and 580 nm are given In Fig. 5(e) clearly shows this ISC conversion process. The singlet decay time constant was fitted with a value of 10 ps and the triplet growth time constant was fit with a value of 8 ps which are within the mutual uncertainty of the fits to the data. The triplet lasts for a long time without further decay on the sub-nanosecond time scale. Unlike the other KP derivatives examined in this study, no carbanion species which feature absorption bands at 607–610 nm was observed in the fs-TA spectra which indicates no photodecarboxylation reaction appears to take place for this molecule.

**Figure 6:**
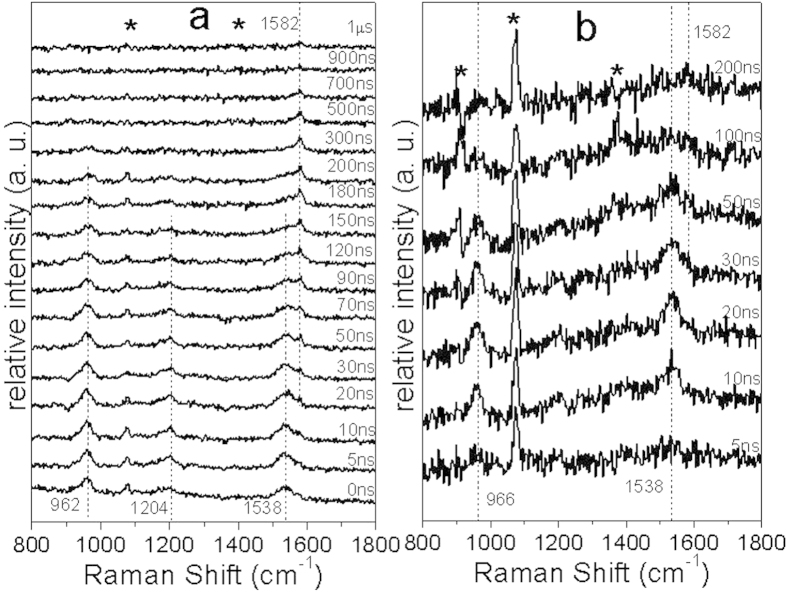
(**a**,**b**) Shown are the ns-TR^3^ spectra of KP-Br and KP-I obtained in a PBS/MeCN = 1:1 by volume solution pH = 7.4 with a 266 nm pump excitation wavelength and a 319.9 nm probe wavelength at various delay times that are inserted next to the spectra. The asterisk (*) marks subtraction artifacts.

Figure 6(a) shows the ns-TR^3^ spectra of KP-Br obtained in a MeCN/PBS = 1:1 by volume solution. KP-Br behaves like benzophenone in an aqueous solution: two species were observed in the spectra which can be attributed to a reaction process that occurs to produce a ketyl radical in an aqueous solution but no photodecarboxylation reaction can be observed. At the very beginning of the acquired spectra at a 0 ns delay time, the first species can be observed with Raman bands at 962, 1204 and 1538 cm^−1^ which can be assigned to the triplet state of KP-Br which decays within 150 ns. A shoulder band at 1582 cm^−1^ gradually grows in intensity up to ~50 ns and then decays over 700 ns. The second species is assigned to a ketyl radical species due to the observation of the characteristic Raman band of 1582 cm^−1^ of the arylphenylketyl (ArPK) radical^21^. Similarly, the ns-TR^3^ spectra of KP-I are shown in Fig. 6(b) and two species were detected and identified as its triplet state and ketyl radical due to the same characteristic Raman bands observed for other benzophenone derivatives’ triplet and ArPK radical assignments respectively^21^. Nevertheless, the signal-to-noise ratio of the intermediate of KP-I appear much weaker and decays much faster than the analogous Raman bands for KP-Br. This shift of the Raman bands indicates that a hydrogen abstraction or other reaction process may take place as a competing reaction with the photodeprotection reaction that results in the release of the functional group from the KP parent structure.

## Discussion

The fs-TA spectra show that the KP-X compounds examined here undergo ISC to their respective triplet states which have absorption bands around 530 nm. The ns-TR^3^ spectroscopy results show that KP-ibuprofen can efficiently undergo a decarboxylation reaction to produce a biradical species with a resonant structure of the benzylic carbanion and this biradical species was directly observed to decay. This indicates that the ibuprofen group is released from the parent KP structure. The model of ketoprofen (KP) connected with the functional group of ibuprofen with an oxygen atom is an efficient platform to release -ibuprofen as it was previously found for the KP-OAc compound. But for the halogen substituent, this situation exhibits interesting changes as the halogen atom nature varies in its electronegativity which may affects the triplet reactivity for driving the functional halogen substituent leaving group. Specifically, the fs-TA of KP-Br indicates that the carbanion species is produced which is the key precursor that is responsible to release the leaving group; yet, for the KP-I system, there are no characteristic transient absorption bands of the carbanion species observed in the PBS solution. For the fingerprint Raman spectra of the KP-Br and KP-I systems, they are similar in that only the triplet and ArPK ketyl radical intermediates were observed in the ns-TR^3^ spectra and no photodecarboxylation reaction related species were observed. Nevertheless, ^1^H and ^13^C NMR photolysis studies indicated that these halogen compounds efficiently release the halogen functional group^20^. Thus, how these halogens are released for the KP-Br and KP-I compounds becomes an interesting question to be answered. We propose that KP-Br and -I may undergo some photodecarboxylation reaction via an excited singlet state and release the Br and I anion groups based on two reasons. First, it has been reported that ketoprofen may undergo a photodecarboxylation reaction via an excited singlet state^24^,^25^. Presumably, the larger halogen substituents change the reactivity of the parent structure and may cause the decarboxylation via the singlet state to become a preferred pathway. Previously, it was reported that a substituent effect may switch on a singlet-character channel for the deprotection reaction^26^. If this singlet-character channel does exist for the KP-Br and -I compounds, the phototrigger reaction for large atom halogen substituent would become much faster than that through the ISC conversion to the triplet channel. It is reasonable that the triplet signal from the ns-TR^3^ may become so weak because of a deprotection reaction that occurs to a more significant extent via the singlet-character reaction channel. Second, the carbon-bromide and carbon-iodine bonds may directly absorb the laser light at wavelengths of 240 nm and 260 nm^27^,^28^ and cleave directly via a singlet pathway which may also subsequently assist the ketoprofen with a carbon dioxide release. If these reactions take place to be a competing pathway, the reason why the KP-Br and KP-I triplet signals are so weak in the ns-TR^3^ spectra becomes self-explanatory. The large element halogen substituent may change the electronic structure of ketoprofen and consequently make the reactivity change so much that it ultimately affects the functional group release pathways. In addition, it is noted that if the species is in the triplet state, the ArPK ketyl radical is observed so that the proton-coupled electron transfer process occurs but not the decarboxylation reaction^29^. This indicates that the triplet state channel for deprotection reaction is not favored for KP-Br and KP-I.

## Methods

### Materials

The three ketoprofen derivatives samples, including ketoprofen-ibuprofen and the halogen derivatives (Bromide and Iodine), were synthesized according to the method reported by Scaiano and co-workers previously^20^ with modifications described in the supporting information with detailed procedures. The structure and purity were confirmed by the related 1H and 13C NMR spectra as shown in the supporting information. The solvent of acetonitrile (MeCN) was used in its spectroscopic grade. The solvent of the phosphate buffered solution (PBS) was prepared with MeCN and water mixed with a volume ratio of 1:1. PBS was prepared by dissolving the phosphorus salts of Na_2_PO_4_ 7.384 g and KH_2_PO_4_ 2.314 g together in 1 liter of deionized water. The final pH value of PBS was 7.4.

### Fs-TA experiments

The femtosecond time-resolved transient absorption (fs-TA) experiments were performed using a commercial femtosecond Ti: Sapphire regenerative amplified laser system and an automated data acquisition system from Ultrafast System. The pulse length of laser is about 120 fs and the repetition rate is 1 kHz. The sample OD was about 1 for a 1 mm path length The pump laser pulse wavelength is 267 nm, the third harmonic of the fundamental 800 nm from the regenerative amplifier. While the probe laser pulse was a white-light continuum from 350 nm to 800 nm generated in a CaF_2_ crystal by using approximately 5% of the amplified 800 nm output from the amplified Ti:sapphire laser system. The probe beam is split into two parts before passing through the sample: one goes through the sample, the other travels directly to the reference spectrometer that monitors the fluctuations in the probe beam intensity. The instrument response time is estimated to be 150 fs. At each of the temporal delay time, the data were averaged for 1 s. The maximum extent of the temporal delay time was 3300 ps for the optical stage used in the experiments. The detailed apparatus and methods for the fs-TA experiments have been discussed previously^23^,^30^.

### Ns-TR^3^ experiments

Nanosecond time-resolved resonance Raman (ns-TR3) measurements were performed using a pump-probe apparatus and methods described previously^21^. Concisely, the pump laser pulse wavelength of 266 nm was generated from the fourth harmonic of a Nd:YAG nanosecond pulsed laser. The probe laser pulse wavelengths of 319.9 nm for ketoprofen derivatives in the MeCN:PBs = 1:1 by volume solutions was generated from the second harmonic of the Nd:YAG nanosecond pulsed laser. The energy of the laser used in the experiments was in a range from 2.5 to 3.5 mJ with a 10 Hz repetition rate. These two lasers were synchronized by a pulse delay generator to electronically control the pump and probe lasers time delay that was monitored by a fast photodiode and 500 MHz oscilloscope. The apparatus time resolution was about 10 ns. The pump and probe laser beams were optically aligned and focused onto a spot of a running sample solution stream so that these two laser pulses were spatially overlapped on the sample solution. The scattered Raman light was collected by using reflective optics into a spectrometer whose grating dispersed the light onto a charge-coupled device (CCD). The Raman signal was accumulated for 30 s by the CCD before being read out and stored to a computer. The spectra included here were obtained by the subtraction of a resonance Raman spectrum with a negative time delay of the probe before the pump -100 ns spectrum from the resonance Raman spectrum obtained with a positive time delay of pump before probe spectrum. The known acetonitrile solvent’s Raman bands were used to calibrate Raman shifts with an approximate accuracy of 5 cm^−1^.

### Density Function Theory (DFT) computation

These calculations were employed to study the properties of ketoprofen for comparison purposes to the experimental data so as to make assignments of the intermediates generated in the excited states. The optimized geometries, vibrational modes and the vibrational frequencies for the different species were obtained from DFT calculations that employed a (U)B3LYP/6-311G** basis set. No imaginary frequency modes were observed at the stationary states of the optimized structures presented here. The default G09 method was used to obtain the Raman spectra to determine the Raman intensity and shifts^31^. A factor of 0.975 were used to scale the calculations of the Raman frequencies by the DFT calculations with (U)B3LYP/6-311G** basis set for comparison with the experimental Raman results in order to achieve a better understanding of the experimental data assignments^32^.

## Additional Information

**How to cite this article**: Liu, M. *et al.* Substituent Effects on the Photodeprotection Reactions of Selected Ketoprofen Derivatives in Phosphate Buffered Aqueous Solutions. *Sci. Rep.* **6**, 21606; doi: 10.1038/srep21606 (2016).

## Supplementary Material

Supporting Information

## Figures and Tables

**Figure 1:**
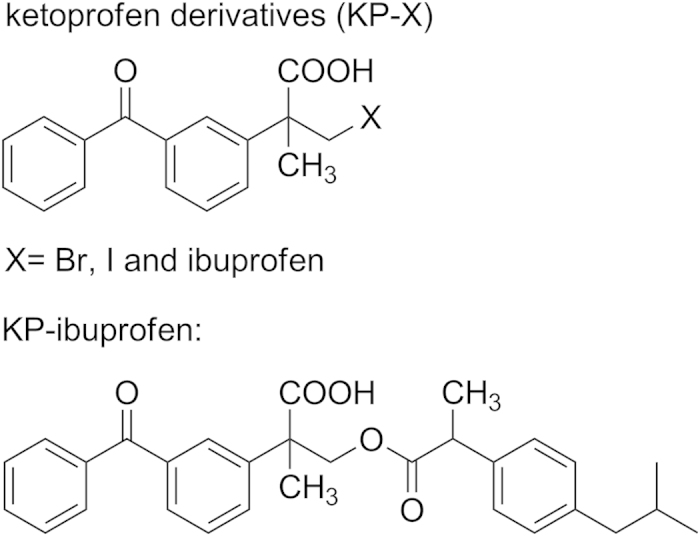
Molecular structures of ketoprofen derivatives.

**Figure 2:**
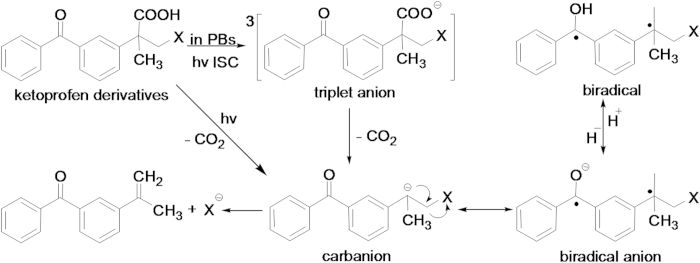
Shown is a proposed mechanism for ketoprofen derivatives deprotection reactions in a phosphate buffered solution.
