# Supplementary material for: Substituent Effects on the Photodeprotection Reactions of Selected Ketoprofen Derivatives in Phosphate Buffered Aqueous Solutions
Source: Sci Rep. 2016 Feb 22;6:21606. doi: 10.1038/srep21606 (PMC4761923; doi:10.1038/srep21606)
Supplement: Supporting Information [file srep21606-s1.doc]

**Supporting Information**

Title: **Substituent Effects on the Photodeprotection Reactions of Selected Ketoprofen Derivatives in Phosphate Buffered Aqueous Solutions**

**Mingyue Liu, Ming-De Li*, Jinqing Huang, Tianlu Li, Han Liu, Xuechen Li and David Lee Phillips***

Department of Chemistry, the University of Hong Kong, Pokfulam Road, Hong Kong

Corresponding Authors: mdli@hku.hk, phillips@hku.hk

Table of Contents

General remarks........................................................................................................................................................S1

Synthesis of 2-[2-(4-isobutylphenyl)propionyloxymethyl]-2-(3-benzoylphenyl)propionic acid.............................S1

Synthesis of 2-bromomethyl-2-(3-benzoylphenyl)propionic acid............................................................................S1

Synthesis of 2-iodomethyl-2-(3-benzoylphenyl)propionic acid................................................................................S2

^1^H and ^13^C NMR spectra of 2-[2-(4-isobutylphenyl)propionyloxymethyl]-2-(3-benzoylphenyl)propionic acid.....S3

^1^H and ^13^C NMR spectra of 2-bromomethyl-2-(3-benzoylphenyl)propionic acid....................................................S4

^1^H and ^13^C NMR spectra of 2-iodomethyl-2-(3-benzoylphenyl)propionic acid.......................................................S5

**General remarks**

The *tert*-butyl 2-hydroxymethyl-2-(3-benzoylphenyl)propionate intermediate was prepared from commercially available 2-(3-benzoylphenyl)-2-methyl propionic acid in 2 steps following published procedures.^20^ The four desired compounds were synthesized from this common intermediate, following published procedures with modifications. The commercially available reagents were used without further purification. The anhydrous THF and toluene were treated with sodium under reflux and freshly distilled before use. The anhydrous DCM was treated with CaH_2_ under reflux and freshly distilled before use. The ^1^H and ^13^C NMR spectra of the products were recorded on Bruker AVANCE 400 spectrometer at 400 MHz and 100 MHz respectively. All NMR data are in accordance with literature results. ^20^

**Synthesis of 2-[2-(4-isobutylphenyl)propionyloxymethyl]-2-(3-benzoylphenyl)propionic acid**

To a stirred solution of *tert*-butyl 2-hydroxymethyl-2-(3-benzoylphenyl)propionate (700 mg, 2.05 mmol, 1 equiv) in anhydrous DCM (10 mL) was added racemic ibuprofen (424 mg, 2.05 mmol, 1 equiv), DCC (846 mg, 4.10 mmol, 2 equiv), and DMAP (49 mg, 0.40 mmol, 0.2 equiv). The mixture was stirred at room temperature over night. The mixture was filtered through a pad of celite to remove DCU, and the filtrate was concentrated. The residue was purified by silica gel column chromatography using *n*-hexane : ethyl acetate 10 : 1 as eluent. The desired product *tert*-butyl 2-[2-(4-isobutylphenyl)propionyloxymethyl]-2-(3-benzoylphenyl)propionate was obtained as colourless oil (1.082 g, 99%), which was directly used in the next step.

To a stirred solution of *tert*-butyl 2-[2-(4-isobutylphenyl)propionyloxymethyl]-2-(3-benzoylphenyl)propionate (1.082 g, 2.04 mmol, 1 equiv) in DCM (5 mL) was added TFA (2.5 mL). The mixture was stirred at room temperature for 16 h. The mixture was diluted with ethyl acetate, thoroughly washed with water and brine, and dried over anhydrous Na_2_SO_4_. The solvent was removed under vacuum, and the residue was purified by silica gel column chromatography using *n*-hexane : ethyl acetate 2 : 1 as eluent. The desired product 2-[2-(4-isobutylphenyl)propionyloxymethyl]-2-(3-benzoylphenyl)propionic acid (mixture of inseparable diastereomers) was obtained as colourless oil (523 mg, 55%). ^1^H NMR (400 MHz, CDCl_3_, selected peaks of major diastereomer): δ = 0.86 (d, *J* = 6.4 Hz, 6H), 1.43 (d, *J* = 6.8 Hz, 3H), 1.54 (s, 3H), 1.77−1.87 (m, 1H), 2.40 (d, *J* = 7.2 Hz, 2H), 3.63−3.69 (m, 1H), 4.42−4.46 (m, 1H), 4.51−4.55 (m, 1H), 7.01 (d, *J* = 7.6 Hz, 2H), 7.09 (d, *J* = 7.6 Hz, 2H), 7.39−7.48 (m, 3H), 7.51 (d, *J* = 8.0 Hz, 1H), 7.58 (t, *J* = 7.2 Hz, 1H), 7.68 (d, *J* = 7.2 Hz, 1H), 7.75 (d, *J* = 6.8 Hz, 2H), 7.80 (s, 1H). ^13^C NMR (100 MHz, CDCl_3_, selected peaks of major diastereomer): δ = 18.0, 20.4, 22.5, 30.3, 45.1, 50.4, 69.0, 127.3, 128.0, 128.5, 128.7, 129.4, 129.7, 130.2, 130.5, 132.8, 137.3, 137.4, 138.0, 139.8, 140.8, 174.3, 178.0, 196.4. All characterization data are in accordance with Scaiano's report.^20^

**Synthesis of 2-bromomethyl-2-(3-benzoylphenyl)propionic acid**

To a stirred solution of *tert*-butyl 2-hydroxymethyl-2-(3-benzoylphenyl)propionate (620 mg, 1.82 mmol, 1 equiv) in anhydrous THF (5 mL) was added CBr_4_ (1.137 g, 3.64 mmol, 2 equiv) and PPh_3_ (957 mg, 3.64 mmol, 2 equiv) under argon. The mixture was stirred at room temperature for 10 h. The mixture was then diluted with ethyl acetate, washed with water and brine, and dried over anhydrous Na_2_SO_4_. The solvent was removed under vacuum, and the residue was purified by silica gel column chromatography using *n*-hexane : ethyl acetate 8 : 1. The desired product *tert*-butyl 2-bromomethyl-2-(3-benzoylphenyl)propionate was obtained as yellowish oil (341 mg, 46%), which was directly used in the next step. The unconverted starting material (158 mg, 26%) was recovered using *n*-hexane : ethyl acetate 2 : 1 as eluent.

To a stirred solution of *tert*-butyl 2-bromomethyl-2-(3-benzoylphenyl)propionate (308 mg, 0.76 mmol, 1 equiv) in DCM (2 mL) was added TFA (1 mL). The mixture was stirred at room temperature for 12 h. The mixture was diluted with ethyl acetate, thoroughly washed with water and brine, and dried over anhydrous Na_2_SO_4_. The solvent was removed under vacuum, and the residue was purified by silica gel column chromatography using *n*-hexane: ethyl acetate 1 : 1 as eluent. The desired product 2-bromomethyl-2-(3-benzoylphenyl)propionic acid was obtained as colourless oil (257 mg, 97%). ^1^H NMR (400 MHz, CDCl_3_): δ = 1.82 (s, 3H), 3.75 (d, *J* = 10.4 Hz, 1H), 4.02 (d, *J* = 10.4 Hz, 1H), 7.46−7.51 (m, 3H), 7.59 (d, *J* = 7.2 Hz, 1H), 7.64 (d, *J* = 8.0 Hz, 1H), 7.73 (d, *J* = 7.2 Hz, 1H), 7.79 (d, *J* = 7.2 Hz, 2H), 7.86 (s, 1H). ^13^C NMR (100 MHz, CDCl_3_): δ = 21.9, 39.7, 51.3, 127.8, 128.2, 128.7, 129.8, 130.1, 130.3, 132.7, 137.1, 137.8, 140.1, 178.6, 196.4. All characterization data are in accordance with Scaiano's report.^20^

**Synthesis of 2-iodomethyl-2-(3-benzoylphenyl)propionic acid**

To a stirred solution of *tert*-butyl 2-hydroxymethyl-2-(3-benzoylphenyl)propionate (358 mg, 1.05 mmol, 1 equiv) in anhydrous toluene (8 mL) was added imidazole (215 mg, 3.15 mmol, 3 equiv), triphenylphosphine (826 mg, 3.15 mmol, 3 equiv), and I_2_ (668 mg, 2.63 mmol, 2.5 equiv) successively under argon. The mixture was stirred under reflux condition for 12 h. After being cooled to room temperature, the mixture was diluted with ethyl acetate, washed with sat. NaHCO_3_ (aq.) and brine, and dried over anhydrous Na_2_SO_4_. The solvent was removed under vacuum, and the residue was purified by silica gel column chromatography using *n*-hexane : ethyl acetate 8 : 1. The desired product *tert*-butyl 2-iodomethyl-2-(3-benzoylphenyl)propionate was obtained as yellowish oil (409 mg, 86%), which was directly used in the next step.

To a stirred solution of *tert*-butyl 2-iodomethyl-2-(3-benzoylphenyl)propionate (409 mg, 0.91 mmol, 1 equiv) in DCM (5 mL) was added TFA (2.5 mL). The mixture was stirred at room temperature for 16 h. The mixture was diluted with ethyl acetate, thoroughly washed with water and brine, and dried over anhydrous Na_2_SO_4_. The solvent was removed under vacuum, and the residue was purified by silica gel column chromatography using *n*-hexane : ethyl acetate 2 : 1 as eluent. The desired product 2-iodomethyl-2-(3-benzoylphenyl)propionic acid was obtained as colourless oil (241 mg, 67%). ^1^H NMR (400 MHz, CDCl_3_): δ = 1.80 (s, 3H), 3.62 (d, *J* = 10.0 Hz, 1H), 3.82 (d, *J* = 10.0 Hz, 1H), 7.44−7.49 (m, 3H), 7.57 (d, *J* = 7.2 Hz, 1H), 7.63 (d, *J* = 8.0 Hz, 1H), 7.72 (d, *J* = 7.2 Hz, 1H), 7.79 (d, *J* = 8.4 Hz, 2H), 7.86 (s, 1H). ^13^C NMR (100 MHz, CDCl_3_): δ = 14.9, 23.9, 50.4, 127.6, 128.3, 128.6, 129.6, 130.1, 130.2, 132.7, 136.9, 137.6, 140.4, 177.9, 196.5. All characterization data are in accordance with Scaiano's report.^21^

**^1^H NMR of 2-[2-(4-isobutylphenyl)propionyloxymethyl]-2-(3-benzoylphenyl)propionic acid**

**^13^C NMR of 2-[2-(4-isobutylphenyl)propionyloxymethyl]-2-(3-benzoylphenyl)propionic acid**

**^1^H NMR of 2-bromomethyl-2-(3-benzoylphenyl)propionic acid**

**^13^C NMR of 2-bromomethyl-2-(3-benzoylphenyl)propionic acid**

**^1^H NMR of 2-iodomethyl-2-(3-benzoylphenyl)propionic acid**

**^13^C NMR of 2-iodomethyl-2-(3-benzoylphenyl)propionic acid**
